# Validation and user experience testing of DataCryptChain: An open-source standard combining blockchain technology with asymmetric encryption for private, secure, shareable, and tamper-proof research data

**DOI:** 10.1371/journal.pdig.0000741

**Published:** 2025-02-24

**Authors:** Jeffrey Michael Franc

**Affiliations:** Department of Emergency Medicine, University of Alberta, Edmonton, Alberta, Canada; Aga Khan University - Kenya, KENYA

## Abstract

DataCryptChain is an open-source standard combining blockchain with advanced encryption ensuring research data remains private, secure, shareable, and tamper-proof. Ability to detect intentional tampering of data was measured, and user experience was evaluated. In this study, simulated datasets were randomized to be tampered with or not tampered with, and detection of tampering was measured. Volunteer’s ability to complete assigned tasks using the software was evaluated. Among 10000 simulated datasets (4436 randomized to tampering) there was 100% sensitivity and specificity for detection. All volunteers successfully installed DataCryptChain and 5/6 completed their tasks. All participants were able to transmit data without ever exposing unencrypted data and with no need to share passwords. Several deficiencies in the user experience were noted. Importantly, the test users felt that although they would be willing to use DataCryptChain in practice, it would need a more user-friendly interface. This study demonstrates a novel algorithm using blockchain and asymmetric encryption that, although previously documented theoretically, has never been published as a working software package. While DataCryptChain has 100% sensitivity and specificity for detecting data tampering, further development is needed to improve the user experience.

## Author summary

Keeping private data secure is of upmost importance for patients and researchers. However, there is no easily accessible and commonly used method to perform research on data and still maintain this security. DataCryptChain is a software tool that researchers can use to ensure data remains private, secure, shareable, and tamper-proof. It combines the technology of blockchain (like that used in Bitcoin and other cryptocurrency) and advanced encryption techniques to do so. In this study, we tested the ability of DataCryptChain to detect if someone was intentionally tampering with research data. The software could detect 100% of the time if data was tampered with. In addition, we tested the ability of scientists to use the software by assigning them some specific tasks to do. Although most scientists (5/6) were able to complete their tasks, most found the software difficult to use. In the end, DataCryptChain is an excellent tool to keep private data secure. However, the tool will need many improvements to make it easier for scientists to use.

## Introduction

In a survey of 600 business and technology executives, 38% stated that poor data quality negatively affects Artificial Intelligence (AI) initiatives [1]. The recent World Health Organization (WHO) document “Regulatory considerations on artificial intelligence for health” - gives highest priority to safeguarding privacy, security, and integrity of healthcare data [2].

In a 2023 review of the challenges in AI for healthcare, Forbes ranked data privacy and security as number one [3]. The Canadian Institute of Health Research recommends that all research limit identifiable data, but admits that de-identification is difficult [4]. This is especially troublesome when entities outside the healthcare system obtain patient or user data for later matching to healthcare data.

DataCryptChain (DCC) is an open-source standard combining blockchain technology with advanced encryption ensuring healthcare research data remains private, secure, shareable, and tamper-proof.

Blockchain is a decentralized digital ledger that records changes so that the record cannot be altered retroactively [5]. All changes to the ledger are identifiable. Cryptocurrency (such as Bitcoin) is a most popular use of blockchain where financial transactions are recorded and cannot be changed retroactively without destroying the integrity of the ledger. Tampering is instantly recognizable. There is no need to keep a central ledger — as a bank might do.

The software tool Git uses blockchain to track changes in computer files [6]. With Git, each version of the files depends on the complete history of the development. Changes to the data must be electronically signed. Git also aids in recovery from accidental changes.

Data encryption “translates data into another form, or code, so that only people with access to a secret key (formally called a decryption key) or password can read it” [7]. Symmetric encryption requires a password for decryption. However, password sharing invites security vulnerabilities. Conversely, asymmetric (public key) encryption targets a specific recipient [8]. Data is encrypted with a public key, and can only be decrypted by the matching private key [9]. Private keys and passwords are never shared. Asymmetric encryption is extremely robust, allowing secure connection to banks and healthcare institutions using the “HTTPS:” protocol.

DataCryptChain combines blockchain and asymmetric encryption to ensure that research data remains private, secure, shareable, and tamper-proof:

1. Private:
  A. Private data, such as patient identifiers, can be encrypted immediately upon acquisition without exposing them to the development team.
  B. Birthdates, addresses, age, date of medical encounter, or other near-identifying data can also be encrypted and excluded from the returned dataset.
2. Secure:
  A. Asymmetric encryption ensures data is readable only by the intended recipient.
  B. Even during a data breach, data is unreadable without the decryption key.
  C. No passwords or decryption keys are shared.
3. Shareable:
  A. DataCryptChain can be used for datasets of any size.
  B. The open-source standard allows developers to create connectors to current statistics software, spreadsheets, or databases.
  C. Data owners can share data with confidence, knowing that the data will be safe at all stages of workflow.
  D. The ease of sharing allows increased collaboration, transparency, and reproducibility.
  E. No central “warehouse” for the data is required.
4. Tamper-proof:
  A. Blockchain technology ensures data integrity by recording and identifying all access and changes to the data.
  B. Intentional tampering of the data will be identifiable and correctable.
  C. Accidental errors can be easily repaired.

Full details of the DCC workflow are described at https://www.datacryptchain.org on the product website. Briefly, DCC functions as follows: First, software is downloaded as a binary or as source code and the command-line executable installed. A new public and secret key pair is created. A new project is initialized, giving a blank .csv (comma separated values) file for data and a ledger file for the blockchain. The user can then add and edit data in the .csv file, and changes are updated into the ledger. To share the data, the ledger is packed into a DataCryptChain (.dcc) file using a recipient’s public key. The encrypted chain can be safely transmitted by any means, such as email. Only the intended recipient can decrypt the file with their secret key, yielding the original ledger and .csv file. The recipient can then edit the .csv, and again update the ledger with the new content. Importantly, every time the data is updated, the blockchain is updated. Any tampering of the data (either intentional or accidentally) will invalidate the blockchain. Validity can be checked at any time through the command line interface.

While the theoretical use of blockchain and asymmetric encryption for data security is compelling, the accuracy of the tool and the acceptance to users is currently unknown.

The primary objective of this study was to measure the sensitivity and specificity of DataCryptChain in detecting intention tampering of data. The secondary objective was to gain feedback on the DataCryptChain user experience (U/X)

## Results

In the unit-testing phase, 10000 simulated research studies were created with data sets ranging from 1 to 2808 records with 1–101 simulated revisions. Of these data sets, 4436 were randomized to be tampered with and were intentionally corrupted by changing the value of a digit. The number of corruptions introduced ranged from 1 to 17 per project. Of the 4436 tampered studies, all were detected by DCC as invalid (sensitivity = 100%). Of the 5564 data sets that were not tampered, DCC labelled all as valid (specificity = 100%). Overall, DataCryptChain showed 100% sensitivity and specificity for detecting these intentional corruptions.

Six users were enrolled in the U/X study phase, four were randomized to user story “Bob” and two were randomized to user story “Alice”. All users were able to install DataCryptChain and 5/6 completed their assigned tasks. Mean overall satisfaction with the product was rated at 4/5.

Of the users who answered the question, 4/ 4 stated that they would be willing to use DCC under operational conditions. Table 1 lists the results of the usability questions. Many users stated that the command-line interface would likely be a major impediment to widespread adoption and recommended a better user interface.

**Table 1:** Usability ratings.

Domain	Median	Minimum	Maximum	Responses
Installation	4	1	10	5
Overall Usability	4	1	10	4
Initialization of a new Project	7.5	4	9	4
Creating a Keyset	8	4	10	5
Editing Data	8	7	10	3
Updating the Ledger	7.5	6	8	4
Packing the DataCryptChain	5	3	5	4
Unpacking the DataCryptChain	6	6	6	1
Extracting from the DataCryptChain	7.5	6	9	2

1 = Very Difficult to Use.

10 = Very Easy to Use.

Throughout the exercise, all participants succeeded in completing the tasks transferring only encrypted data. At no time was unencrypted data sent by email. In addition, no passwords were exchanged.

## Discussion

This study indicates that the combination of blockchain technology with currently available cryptographic algorithms has a 100% sensitivity and specificity for detecting data tampering. Furthermore, the study demonstrates that users can create datasets and transfer them by email without ever exposing unencrypted data and without the need for password sharing.

Despite the WHO mandate, there is no current standard for ensuring research data security. Data is often stored and transmitted unencrypted by researchers. Costly data breaches are common. In July 2013, four computers were stolen from a building and unencrypted data for 4 million patients was compromised, leading to a $5.55 million fine [10]. In 2020, the AI development company Babylon suffered a data breach exposing videos of patient encounters [11].

Data sharing is the practice of making data available to other research stakeholders such as other investigators, research subjects, and the broader public [12]. The WHO rates the availability of good quality, clinically relevant datasets as a key challenge in the development of AI in healthcare [2]. The lack of shareability of data is also a key impediment to making data science reproducible [13]. The recent fall of the AI healthcare company Babylon was at least partially due to the lack of transparency and sharing of data [11]. Unfortunately, there is no standard format for sharing private research data. This is complicated by the fact that researchers do not all use the same analysis software — any successful solution must be compatible with a number of different software applications.

Errors in data occur — both accidentally and deliberately [14]. In a recent survey, 2% of scientists admitted to falsifying or fabricating data [15]. Up to 35% of scientific journal retractions are due to misconduct [16]. This is more common when scientists target high-impact journals [17]. Such tampering can be harmful: the common fear of autism caused by vaccination is largely based on falsified data [18]. Current statistical techniques to detect data fraud are marginal [19].

Although blockchain clearly holds promise in data management, little practical research has been performed. Mamoshina (2018) stated that blockchain holds promise to “resolve the challenges faced by regulators and return control over personal data to the individuals” [5]. Blockchain has been recognized as a way to provide secure storage of data with an integrity guarantee [20]. Furthermore, blockchain could be standardized and be therefore interoperable across various systems [9]. Data sharing could be facilitated by efficient interoperability [20]. Blockchain has also been recognized for its traceability [9]. In addition, a theoretical construct, using smart contracts on the Ethereum platform was designed as a way to improve the transparency of data management in clinical trials [21]. However, a 2020 scoping review found only two articles focused on blockchain for research data — both provided theoretical constructs only [22].

Likewise, despite its potential, asymmetric encryption is seldom used for research data. Das et al. have detailed a theoretical model to protect patient identifiers from wearable devices [23]. Wirth et al. have postulated a cryptographically secure multi-party protocol to allow analysis or encrypted data [24]. This could be done using multi-party computation protocols or homomorphic encryption — previously used for the Boston wage equity initiative [25].

While cloud-based computing storage is often offered to increase data security, it has several shortcomings. Firstly, in contract to DCC, cloud solutions are expensive, and may be unreachable for researchers who work independently, at smaller institutions, or in lower income settings. Secondly, these solutions frequently force researchers to use new platforms, increasing the workflow burden. Finally, security breaches of cloud providers are increasingly common [26].

### Strengths and limitations

The strength of this study is in the proof of concept of a novel algorithm using blockchain and asymmetric encryption that, although previously documented theoretically, has never been published as a working software package.

There are several limitations in the study. Firstly, DCC was not tested against large data sets such as genomics or images. Secondly, the U/X study involved only six users, all of whom were computer experts. Thus, although most users in this study completed their assigned task, the same success should not be expected from all researchers. Finally, the cryptographic integrity of the DCC files was not tested, as the cryptographic techniques have an established safety record.

### Significance

This study suggests that DataCryptChain could become an accurate addition to researcher’s workflow if the user interface were improved. User interface improvements could include simplification of the installation process; better integration with existing databases and spreadsheets; simplified management of cryptography keys; and automated updating and validating of the ledger.

Further validation will be needed to ensure the integrity and usability of the blockchain with larger data sets. In addition, the existing tool does not involve user specific cryptographic signing of the blockchain, which should be added to ensure better tracking of data errors or data tampering.

### Conclusion

DataCryptChain has the potential to keep research data private, secure, shareable, and tamper-proof by combining blockchain and asymmetric encryption. The existing tool has 100% sensitivity and specificity for detecting data tampering but will need further development to ensure the tool remains user-friendly and to encourage widespread adoption.

## Materials and methods

The Health Research Ethics Board of the University of Alberta approved the study (Pro00141418).

In the unit-testing phase, 10000 simulated research projects were analyzed. For each iteration, a data set was created and repeatedly modified, encrypted with DataCryptChain and then decrypted. Each iteration was randomized to be tampered or not tampered, and sensitivity and specificity to detect the tampering was calculated. Sensitivity was defined as the percentage of projects that were intentionally tampered with that were appropriately detected by DCC as invalid. Specificity was defined as the percentage of projects that were not tampered with that were appropriately labelled by DCC as valid.

A convenience sample of test users was recruited from personal contacts of the authors. Participants were identified specifically for their computing science experience. Participants were contacted by email asking their consent to take part in a research study for a new data management software.

U/X testing participants were randomly assigned to one of two user stories. User story “Alice” invited the user to install the DCC software, create a DCC keyset, send their public key by email requesting a dataset, and then unpack the DataCryptChain to view the .csv (S1 Appendix). Task completion of this user story was defined as the ability to identify a single data point (the name of a white dog) in the dataset. User story “Bob” invited the user to install the DCC software, create a new DCC project, encrypt it with Bob’s public key, then send the completed .dcc file by email (S2 Appendix). Task completion for this user story was defined as successful email transmission of an encrypted DataCryptChain file that could be decrypted using the original public key paired to Bob’s secret key.

The user story cards asked participants to complete an online satisfaction survey after completing their assigned tasks (S3 Appendix).

## Supporting information

S1 Appendix User Story Alice. (PDF)

S2 Appendix User Story Bob. (PDF)

S3 Appendix User Experience Survey. (PDF)

## References

[1] Woodie A. Room for improvement in data quality, report says; 2020 [cited 2024 Jul 15]. Available from: https://www.datanami.com/2020/01/23/room-for-improvement-in-data-quality-report-says/

[2] World Health Organization. Regulatory considerations on artificial intelligence for health. 2023 [cited 2024 Jul 15]. Available from: https://iris.who.int/handle/10665/373421

[3] Rosen H. Top five opportunities and challenges of AI in Healthcare. Forbes. 2023 [cited 2024 Jan 21]. Available from: https://www.forbes.com/sites/forbesbusinesscouncil/2023/02/07/top-five-opportunities-and-challenges-of-ai-in-healthcare/?sh=605a07c22805

[4] Canadian Institutes of Health Research Best Practices for Protecting Privacy in Health Research. 2005 [cited 2024 Jan 15]. Available from: https://cihr-irsc.gc.ca/e/documents/et_pbp_nov05_sept2005_e.pdf

[5] Mamoshina P, Ojomoko L, Yanovich Y, Ostrovski A, Botezatu A, Prikhodko P, et al. Converging blockchain and next-generation artificial intelligence technologies to decentralize and accelerate biomedical research and healthcare. Oncotarget. 2018;9(5):5665–90. doi: 10.18632/oncotarget.22345 29464026 PMC5814166

[6] Spinellis D. Git. IEEE Softw. 2012;29(3):100–1. doi: 10.1109/ms.2012.61

[7] De Groot J. What is data encryption? (Definition, Best Practices, and More). 2023 [cited 2024 Jul 15]. Available from: https://www.digitalguardian.com/blog/what-data-encryption

[8] Sukte C, Emmanuel M, Deshmukh R. Novel Approach for improving security and confidentiality of PHR in cloud using public key encryption. In: Rajakumar G, Du K, Vuppalapati C, Beligiannis GN, editors. Intelligent communication technologies and virtual mobile networks lecture notes on data engineering and communications technologies. Singapore: Springer; 2022.

[9] Yaqoob I, Salah K, Jayaraman R, Al-Hammadi Y. Blockchain for healthcare data management: opportunities, challenges, and future recommendations. Neural Comput Appl. 2021;34(14):11475–90. doi: 10.1007/s00521-020-05519-w

[10] Ouellette P. Advocate Medical Group endures massive data breach. 2013 [cited 2024 Jul 14]. Available from: https://healthitsecurity.com/news/advocate-medical-group-endures-massive-data-breach

[11] Browne G. The fall of Babylon is a warning for AI unicorns. 2023 [cited 2024 Jul 15]. Available from: https://www.wired.com/story/babylon-health-warning-ai-unicorns/

[12] Data sharing national library of medicine. [cited 2024 Jul 15]. Available from: https://www.nnlm.gov/guides/data-glossary/data-sharing

[13] Peng R. The reproducibility crisis in Science: a statistical counterattack. Significance. 2015;12(3):30–2. doi: 10.1111/j.1740-9713.2015.00827.x

[14] Twenter P. Drug researcher charged in $16M case. 2024 [cited 2024 Jul 15]. Available from: https://www.beckershospitalreview.com/legal-regulatory-issues/drug-researcher-charged-in-16m-case.html

[15] Fanelli D. How many scientists fabricate and falsify research? A systematic review and meta-analysis of survey data. PLoS One. 2009;4(5):e5738. doi: 10.1371/journal.pone.0005738 19478950 PMC2685008

[16] Wray KB, Andersen LE. Retractions in science. Scientometrics. 2018;117(3):2009–19. doi: 10.1007/s11192-018-2922-4

[17] Steen RG. Retractions in the scientific literature: do authors deliberately commit research fraud? J Med Ethics. 2011;37:113–7. 21081306 10.1136/jme.2010.038125

[18] Godlee F, Smith J, Marcovitch H. Wakefield’s article linking MMR vaccine and autism was fraudulent. BMJ. 2011;342:c7452. doi: 10.1136/bmj.c7452 21209060

[19] George SL, Buyse M. Data fraud in clinical trials. Clin Investig (Lond). 2015;5(2):161–73. doi: 10.4155/cli.14.116 25729561 PMC4340084

[20] Zhang R, Xue R, Liu L. Security and privacy for healthcare blockchains. IEEE Trans Serv Comput. 2022;15(6):3668–86. doi: 10.1109/tsc.2021.3085913

[21] Nugent T, Upton D, Cimpoesu M. Improving data transparency in clinical trials using blockchain smart contracts. F1000Res. 2016;5(5):2541. doi: 10.12688/f1000research.9756.1 28357041 PMC5357027

[22] Hasselgren A, Kralevska K, Gligoroski D, Pedersen SA, Faxvaag A. Blockchain in healthcare and health sciences-A scoping review. Int J Med Inform. 2020;134:104040. doi: 10.1016/j.ijmedinf.2019.104040 31865055

[23] Das S, Namasudra S. A novel hybrid encryption method to secure healthcare data in IoT-enabled healthcare infrastructure. Comput Electr Eng. 2022;101:107991. doi: 10.1016/j.compeleceng.2022.107991

[24] Wirth FN, Meurers T, Johns M, Prasser F. Privacy-preserving data sharing infrastructures for medical research: systematization and comparison. BMC Med Inform Decis Mak. 2021;21(1):242. doi: 10.1186/s12911-021-01602-x 34384406 PMC8359765

[25] Boston Co. City of Boston: closing the wage gap. 2013 [cited 2024 Jan 21]. Available from: https://www.cityofboston.gov/images_documents/Boston_ClosingtheWageGap_InterventionsReport_tcm3-41353.pdf

[26] Novet J. AT&T’s massive data breach deepens crisis for Snowflake seven weeks after hack was disclosed. 2024 [cited 2024 Jan 21]. Available from: https://www.cnbc.com/2024/07/12/snowflake-shares-slip-after-att-says-hackers-accessed-data.html

