# Supplementary material for: Validation and user experience testing of DataCryptChain: An open-source standard combining blockchain technology with asymmetric encryption for private, secure, shareable, and tamper-proof research data
Source: PLOS Digit Health. 2025 Feb 24;4(2):e0000741. doi: 10.1371/journal.pdig.0000741 (PMC11849895; doi:10.1371/journal.pdig.0000741)
Supplement: S1 Appendix — (PDF) [file pdig.0000741.s001.pdf]

Thank-you for agreeing to be a part of the DataCryptChain User Experience (U/X) study. The goal of the this study is to assess the ability of participants to complete the User Story below using only information available on the product website at https://www.datacryptchain.org . The study is designed to test the User Experience of the DataCryptChain software and the adequacy of the online documentation.

USER STORY: ALICE

Alice is a veterinary researcher who is studying poodles. Your task is to obtain the *Poodles* DataCryptChain from her and open the dataset by performing the following:

1. Download and install the DataCryptChain software.
2. Create a new keyset for yourself.
3. Send Alice an email to alice@datacryptchain.org containing your public key and requesting the *Poodles* DataCryptChain.
4. When you receive the *Poodles* DataCryptChain unpack it to view the data.
5. Finally, please complete the U/X survey at: https://survey.stat59.com/index.php/XXXX

We ask you to please try to complete the tasks above using only the information available on the DataCryptChain website. If you are unable to complete the tasks above, you can stop the study at any time. We do, however, ask you still complete the U/X survey.
