# Supplementary material for: Validation and user experience testing of DataCryptChain: An open-source standard combining blockchain technology with asymmetric encryption for private, secure, shareable, and tamper-proof research data
Source: PLOS Digit Health. 2025 Feb 24;4(2):e0000741. doi: 10.1371/journal.pdig.0000741 (PMC11849895; doi:10.1371/journal.pdig.0000741)
Supplement: S2 Appendix — (PDF) [file pdig.0000741.s002.pdf]

Thank-you for agreeing to be a part of the DataCryptChain User Experience (U/X) study. The goal of the this study is to assess the ability of participants to complete the User Story below using only information available on the product website at https://www.datacryptchain.org . The study is designed to test the User Experience of the DataCryptChain software and the adequacy of the online documentation.

USER STORY: BOB

You are a researcher doing a project on pets. Bob is a statistician who will help with the data analysis. Your task is to create a small DataCryptChain dataset called Pets and send it to Bob.

1. Download and install the DataCryptChain software.
2. Initialize a new DataCryptChain project called *Pets*.
3. Add some synthetic data including name, species, and color to the DataCryptChain. You can simply make-up your own data.
4. Send Bob an email to bob@datacryptchain.org to obtain his public key.
5. Package the DataCryptChain using Bob's key and email the *Pets* DataCryptChain back to Bob.
6. Finally, please complete the U/X survey at: https://survey.stat59.com/index.php/XXXX

We ask you to please try to complete the tasks above using only the information available on the DataCryptChain website. If you are unable to complete the tasks above, you can stop the study at any time. We do, however, ask you still complete the U/X survey.
