# Supplementary material for: Validation and user experience testing of DataCryptChain: An open-source standard combining blockchain technology with asymmetric encryption for private, secure, shareable, and tamper-proof research data
Source: PLOS Digit Health. 2025 Feb 24;4(2):e0000741. doi: 10.1371/journal.pdig.0000741 (PMC11849895; doi:10.1371/journal.pdig.0000741)
Supplement: S3 Appendix — (PDF) [file pdig.0000741.s003.pdf]

DataCryptChain U/X Survey

Thank-you for participating in the DataCryptChain User Experience Testing study. Please answer the following questions based on your experience using the DataCryptChain software to complete your assigned user story.

Your responses will be anonymous. You do not need to give your name or identifiers.

This survey should take 5-10 minutes to complete.

Your information is held by STAT59 under strict privacy (https://www.stat59.com/about/privacy) and security (https://www.stat59.com/about/security) policies. By completing this survey, you are consenting to these policies.

There are 10 questions in this survey.

Overall Rating

How would you rate the DataCryptChain software overall? *

Please choose **only one** of the following:

- ◯ 1
- ◯ 2
- ◯ 3
- ◯ 4
- ◯ 5

Usability

Please rate the usability of the following components. Where 1 is "very difficult to use" and 10 is "very easy to use"

Please choose the appropriate response for each item:

	1	2	3	4	5	6	7	8	9	10
**Installation of the Software**	○	○	○	○	○	○	○	○	○	○
**Initialiizing a New Project**	○	○	○	○	○	○	○	○	○	○
**Creating a Keyset**	○	○	○	○	○	○	○	○	○	○
**Editing Data**	○	○	○	○	○	○	○	○	○	○
**Updating the Ledger**	○	○	○	○	○	○	○	○	○	○
**Packing the Ledger**	○	○	○	○	○	○	○	○	○	○
**Unpacking the Ledger**	○	○	○	○	○	○	○	○	○	○
**Extracting the CSV file**	○	○	○	○	○	○	○	○	○	○

User Story

Which user story did you complete?

❗ Check all that apply

Please choose **all** that apply:

- ☐ Alice
- ☐ Bob

If you completed the User Story "Alice", what was the name of the white poodle?

Please write your answer here:

User Data

Which Operating System did you use?

Check all that apply

Please choose **all** that apply:

- [ ] Windows
- [ ] MacOS
- [ ] Linux
- [ ] Other:

How did you install DataCryptChain?

Check all that apply

Please choose **all** that apply:

- [ ] From Binary
- [ ] From Source

What application did you use to edit / view the .csv data?

❗ Check all that apply

Please choose **all** that apply:

- ☐ Microsoft Excel
- ☐ LibreOffice
- ☐ OpenOffice
- ☐ Other: ______

Future Use

Would you be willing to use DataCryptChain under operational conditions?

Please choose **only one** of the following:

- ○ Yes
- ○ No

Your experience as a test user is essential to use. Please use this space to provide any comment, criticisms, or suggestions that might improve the software.

Please write your answer here:

Please provide any other remarks about the DataCryptChain software or the research project.

Please write your answer here:

Submit your survey.

Thank you for completing this survey.
